# Specialized Delirium Care Environments in Hospitalized Older Adults: A Systematic Review

**DOI:** 10.3390/geriatrics11050122

**Published:** 2026-09-04

**Authors:** Henri Perrin, Giulio Mastria, Alberto Garcia Manjon, Patrizia D’Amelio

**Affiliations:** 1 Geriatrics Unit, Department of Internal Medicine, Lausanne University Hospital, 1012 Lausanne, Switzerland; alberto.garcia-manjon@chuv.ch (A.G.M.); patrizia.damelio@chuv.ch (P.D.); 2 Nuffield Department of Clinical Neurosciences, University of Oxford, Oxford OX3 9DU, UK

**Keywords:** delirium, non-pharmacological, room design, older patients

## Abstract

**Purpose:** Delirium is a frequent and serious condition in older patients, associated with severe adverse outcomes and lacking proven pharmacological treatments. Non-pharmacological multicomponent strategies are recommended, and specialized delirium care environments (e.g., delirium room, delirium unit, psychogeriatric unit) have been proposed as a potential strategy to improve the management of delirium in hospitalized older adults. Our objective was to provide the first systematic review and pooled analysis on the subject, synthesizing the characteristics of specialized delirium environments in the care of delirium and their association with clinical outcomes. **Methods:** A systematic search of MEDLINE, Cochrane, and EMBASE identified studies on patients aged 65 years and older with delirium or related acute confusional states admitted to specialized delirium care environments. Study quality was assessed using Cochrane’s risk of bias tools, and exploratory pooled analyses were conducted when at least three studies reported the same outcome. **Results:** Nine studies, reported across 15 publications and including 2226 patients, met the inclusion criteria. Interventions were heterogeneous in structure and content, but most combined delirium-oriented staff training, enhanced surveillance, environmental adaptation, and multicomponent non-pharmacological care. Comparator groups were also diverse and included standard wards, earlier versions of specialized care models, indirect admission pathways, and non-delirious controls. The narrative synthesis suggested that specialized delirium care environments were most consistently associated with shorter delirium duration, more favorable discharge outcomes, lower physical restraint use, and better functional recovery, while findings for length of stay, falls, psychotropic drug use, and mortality were less consistent. Exploratory pooled analyses suggested a favorable direction of effect for several outcomes, particularly discharge destination and mortality, but pooled estimates were not statistically significant and should not be interpreted as definitive evidence of efficacy. All included studies were judged to be at a high or critical risk of bias. **Conclusions:** Specialized delirium care environments appear promising for the management of delirium in hospitalized older adults, particularly for outcomes closely related to day-to-day delirium care. However, the current evidence base is limited by substantial heterogeneity in intervention models, comparator groups, and outcome definitions, as well as by a high risk of bias across studies. These findings support further evaluation of specialized delirium care models, but do not yet allow firm conclusions regarding their effectiveness.

## 1. Introduction

Delirium is a neuropsychiatric syndrome characterized by an acute disturbance in attention and awareness, with a fluctuating course, developing over a short period of time not better explained by a pre-existing neurocognitive disorder [1]. It is a serious condition amongst older adults, affecting 8–17% of older adults presenting to the emergency department and developing in 20 to 29% of hospitalized older patients [2]. Its etiology is multifactorial, involving predisposing factors such as older age, comorbidities, and exposure to psychotropic substances or medications, along with precipitating events including major trauma or surgery. These factors contribute to transient disruption of neuronal function through alterations in neurotransmitters and neuronal networks [2,3]. Delirium is associated with significant adverse outcomes, including prolonged hospitalization, higher mortality, long-term cognitive decline, and increased risk of institutionalization [4,5,6]. The broader geriatric context is also important, as hospitalized older adults frequently present with multiple chronic conditions which may increase vulnerability to delirium and add complexity to its clinical management [7].

Given its clinical impact, prevention and optimal management of delirium are major priorities in geriatric care. Current guidelines strongly recommend multicomponent non-pharmacological interventions as the cornerstone of both prevention and management [8,9]. When delirium occurs, treatment strategies must be applied to shorten its duration and mitigate its complications. Due to its heterogeneous presentation and multifactorial pathophysiology, no single standardized approach exists, and pharmacological treatments lack convincing evidence of effectiveness. No clear consensus exists on the efficacy of pharmacological interventions; accordingly, guidelines prioritize non-pharmacological strategies such as re-orientation, early mobility, cognitive stimulation, sleep enhancement, music therapy, and light therapy [10,11,12,13].

To deliver these interventions more effectively, several specialized inpatient care models have emerged over time. Although described using heterogeneous terminology such as delirium rooms, delirium units, psychogeriatric units, geriatric monitoring units, and specialist medical and mental health units, these models share a common goal: systematically improving delirium care through structured, multidisciplinary, non-pharmacological approaches [14,15]. Despite growing interest, the evidence supporting these specialized environments remains dispersed, and variations in terminology, design, and implementation complicate interpretation across studies.

The aim of this study was to provide the first systematic review and exploratory pooled analysis assessing the characteristics of specialized delirium care environments (SDCEs) and exploring their association with clinical outcomes in older hospitalized patients. By synthesizing the available evidence, this review seeks to support the development of evidence-based approaches for implementing specialized delirium care environments and improving delirium management in clinical practice.

## 2. Methodology

### 2.1. Study Design

A systematic review was conducted according to the Preferred Reporting Items for Systematic Reviews and Meta-analysis (PRISMA) statement [16]. The review protocol is published on the PROSPERO database (PROSPERO 2023 CRD42023396118, https://www.crd.york.ac.uk/PROSPERO/view/CRD42023396118, accessed on 31 august 2026).

### 2.2. Inclusion and Exclusion Criteria

We included observational and intervention studies, clinical trials, prospective or retrospective controlled cohort studies, and case–control studies published in English. Reviews, case reports, study protocols, and reports not written in English were excluded.

### 2.3. Search Strategies

The research question was formulated by the following PICO (Participants, Intervention, Comparison, Outcomes) strategy.

- Participants: hospitalized adults 65 years or older affected by delirium or acute confusional state.
- Intervention: hospitalization in a specialized delirium care environment (e.g., delirium room, delirium unit, psychogeriatric unit).
- Comparison: not required.
- Outcomes: delirium duration, length of stay, complications, pharmacological and physical restraints, discharge destination, and caregiver satisfaction.

Systematic searches were conducted on MEDLINE, EMBASE, and Cochrane Library databases using terms related to Delirium and Delirium Room/Units. Both free words and MeSH terms were used. Searches were limited to English-language publications available before the 1st of February 2023.

A supplementary hand-search of citations from included studies was performed. An additional titsearch using the same search strategy was conducted on 1st of April 2026 and did not identify any further studies fulfilling the inclusion criteria. The search strings are available in Supplementary Table S1.

Given the variability of terminology and service design, we included any specialized inpatient models focused on delirium or related acute confusional states, including delirium rooms, delirium units, psychogeriatric units, and dedicated geriatric delirium care pathways. Studies with non-equivalent comparator or broader population definitions were retained to characterize the evidence base but interpreted with caution in pooled analyses.

### 2.4. Study Selection

Two reviewers (H.P., A.G.) independently screened titles and abstracts using the Rayyan^®^ platform (available at Rayyan–Intelligent Systematic Review–Rayyan, https://www.rayyan.ai). Disagreements were resolved through discussion; if unresolved, a third reviewer (PDA) was consulted.

### 2.5. Data Extraction and Data Synthesis

Data extraction was performed independently by two reviewers (H.P., G.M.) and cross-verified. Extracted items followed Cochrane’s Handbook recommendations (Version 6.3, Section 5.3) and were organized in a pre-structured Excel spreadsheet. For studies appearing in multiple reports, demographic data and main outcomes were extracted only once; additional reports were used for relative sub-analysis when relevant. Only data pertinent to the objectives of this review were included. Extracted data were synthesized according to Cochrane’s Handbook (Version 6.3, Chapter 9) and table summaries were generated.

### 2.6. Risk of Bias Assessment

The risk of bias was independently assessed by two reviewers (H.P., G.M.), using Cochrane’s risk of bias tools. Randomized controlled trials were evaluated with the RoB-2 tool, and non-randomized studies with the ROBINS-I tool. Results of the assessment were synthesized using Cochrane’s robvis tool.

### 2.7. Data Analysis

An exploratory pooled analysis was performed for outcomes reported by at least three studies. Two studies were excluded from pooling due to a lack of patients with delirium in the control group [17,18]. One additional study [19] was included only for dichotomous outcomes due to missing variance data for continuous measures. For continuous outcomes, effect sizes were calculated; for dichotomous outcomes, odds ratios were estimated. When studies used different formats for the same outcome (continuous vs. dichotomous), odds ratios were converted to effect sizes using established formulae [20]. In studies with two control groups [21,22], control data were combined. Random-effects models were used to account for expected heterogeneity. Sensitivity and heterogeneity analyses were carried out for each outcome. Sensitivity analyses were performed using a leave-one-out approach, in which each study was sequentially removed from the pooled analysis. Given the small number of studies, results were interpreted by assessing whether exclusion of individual studies changed the direction of effect. Forest plots summarize the pooled results. Given the substantial clinical and methodological heterogeneity across studies, all pooled analyses were treated as exploratory.

## 3. Results

### 3.1. Systematic Review

#### 3.1.1. Record Selection

The database search conducted on 1st February 2023 yielded 50 records. After removing duplicates, the titles and abstracts of 29 records were screened, of which 11 reports met initial eligibility criteria. Full-text reviews resulted in the inclusion of nine reports.

**Figure 1:**
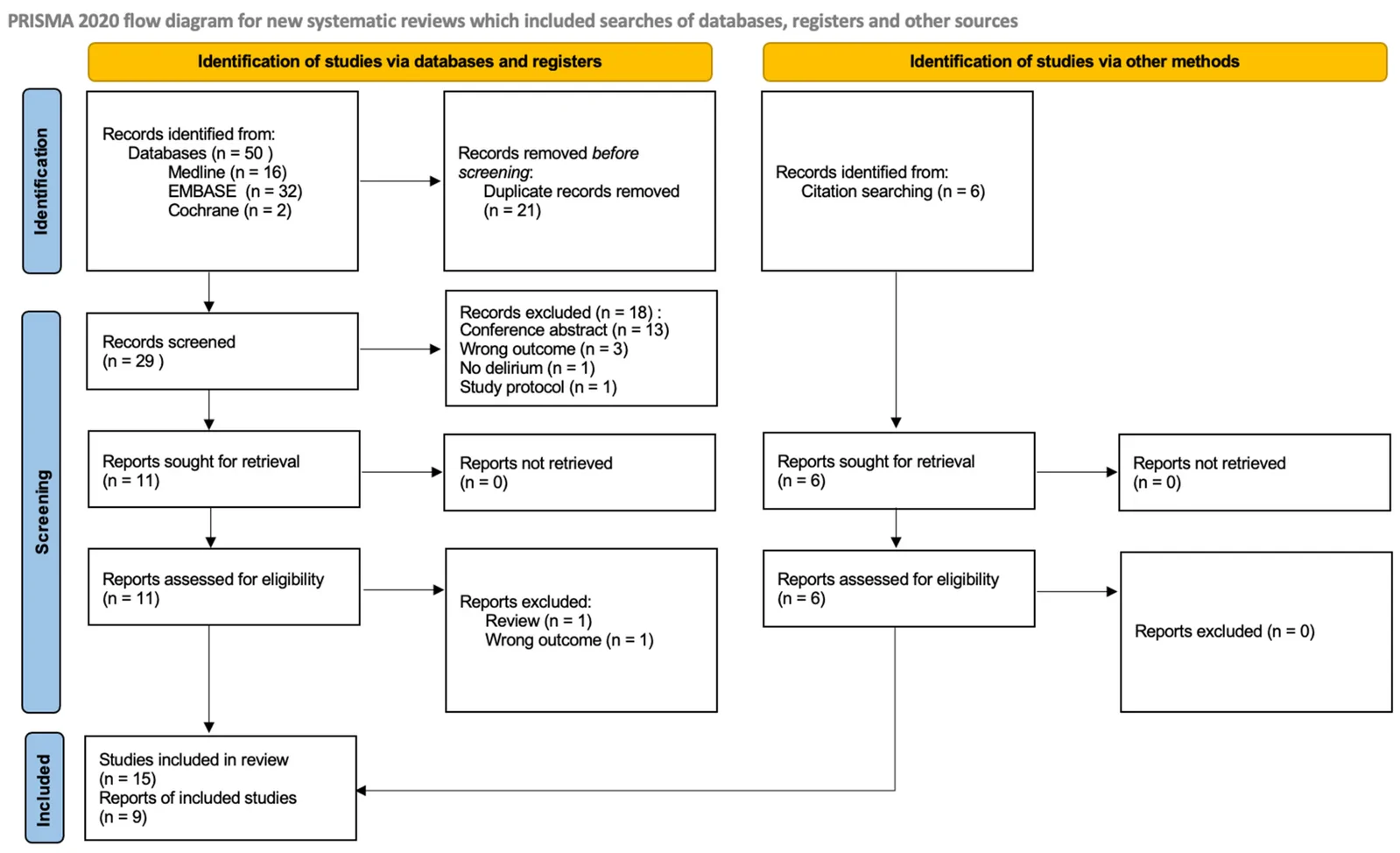
PRISMA protocol flow diagram.

Citation screening identified an additional six reports, increasing the total to 15 reports representing nine studies. Exclusion reasons and the selection flow diagram (PRISMA 2020) are presented in Figure 1.

The studies were classified as retrospective (two studies), before–after (three studies), RCTs (1 study), non-randomized cohort studies (two studies), and mixed (RCT and retrospective).

Table 1 displays a summary of the studies’ characteristics and main findings.

#### 3.1.2. Demographics, SDCE Characteristics, and Intervention Features

A total of 2226 patients were evaluated in the included studies, of which 1321 were part of the intervention group and 905 were part of the control group. The mean age was 83.4 (SD 7.8), and the female-to-male ratio was 3:2. The included studies spanned four continents and showed substantial variation in patient characteristics. Most participants had existing cognitive impairment (64%), and 61% were admitted from home. Delirium diagnosis was most frequently established using the CAM method. Not all the studies included complete demographic data. Participants’ characteristics for intervention and control groups are summarized in Supplementary Tables S3 and S4, respectively.

Intervention models differed considerably. The number of delirium-dedicated beds ranged from 4 beds within a single room to 28 within a unit. All the studies implemented staff interventions, including increased surveillance (through video monitoring and/or increased nurse-to-patient ratio), delirium-specific staff training, and a multidisciplinary care approach. Environmental features commonly included safe room design, reorientation strategies (clock and calendar and/or personal items), and adequate sensory optimization (noise reduction, appropriate lighting). Many studies recommended avoidance or minimization of psychotropic use and favoring antipsychotics over sedatives when needed. Non-pharmacological interventions aligned with delirium care guidelines, such as the HELP program [23], and included avoidance of physical restraints, early mobilization, cognitive stimulation, sleep-supportive measures, and management of modifiable delirium risk factors. Intervention components are summarized in Table 2.

Control groups varied widely. In most studies (60%), controls were delirious patients hospitalized in a standard ward unit. Others used historical controls: retrospective data of patients receiving a different type of intervention (often an earlier version of the DR) [24], patients indirectly admitted to the SDCE (i.e., with an initial stay in a standard ward followed by a transfer to an SDCE) [25], patients without delirium [18], or no control group [17]. These differences were considered when interpreting pooled results.

### 3.2. Outcomes

**Figure 2:**
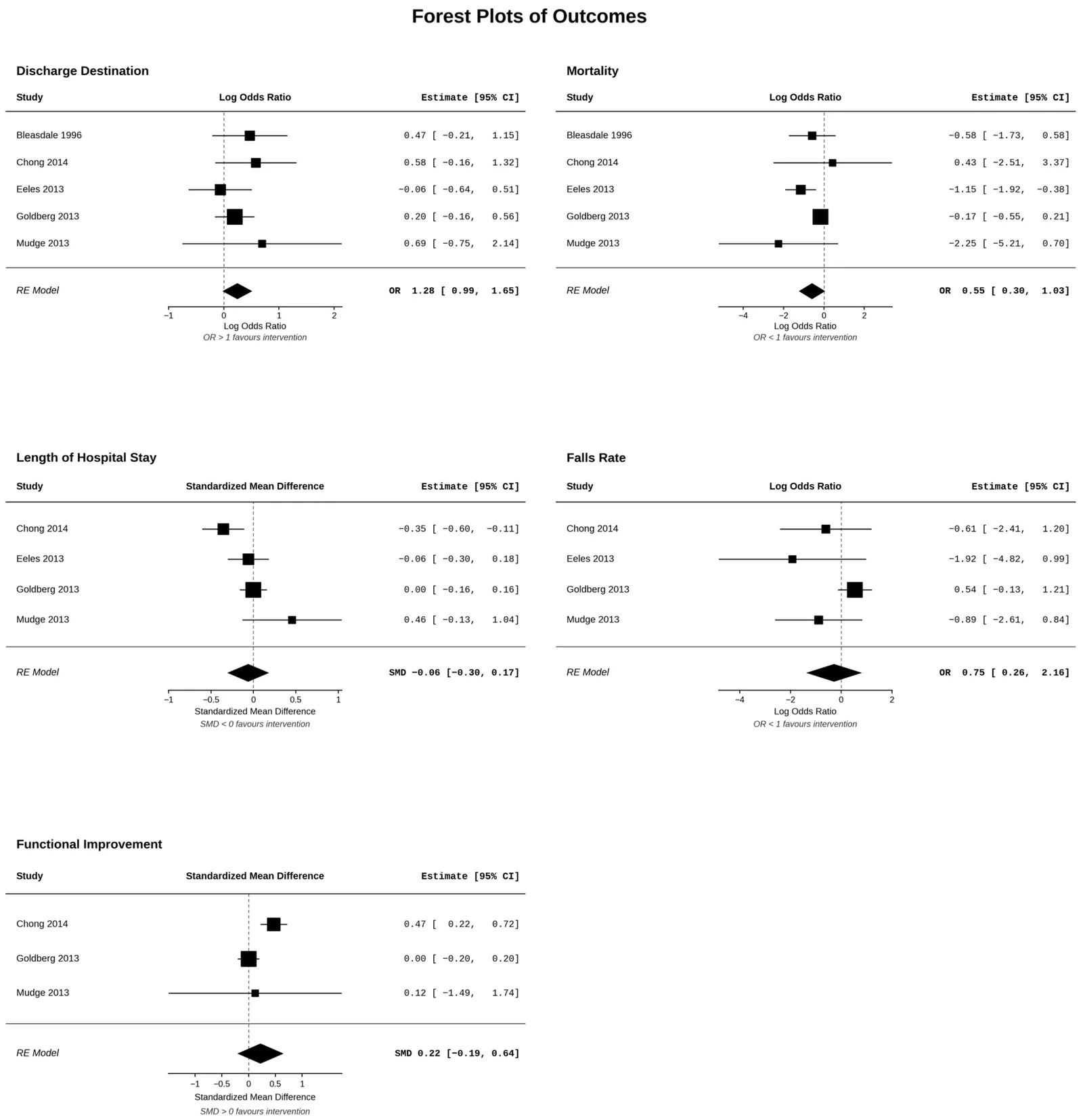
Forest plots for each measured outcome [19,21,22,26,27]. Squares represent point estimates for individual studies, with square size proportional to study weight; horizontal lines indicate 95% confidence intervals; diamonds represent the pooled random-effects estimate.

Results are presented by outcome; for each outcome, we first provide a narrative synthesis that considers the type of intervention and comparator, followed, where feasible, by an exploratory pooled analysis. Forest plots for exploratory pooled analysis are shown in Figure 2.

#### 3.2.1. Delirium Duration

Delirium duration was assessed in four studies [21,24,25,26]. Three studies measured mean duration of delirium in days, while one study reported the proportion of patients with persistent delirium when discharged. Two studies compared SDCEs versus standard wards, with one study showing significantly shorter delirium duration and one study showing mixed results, with significantly shorter duration when using a retrospective control group and a small non-significant increase in duration when using a prospective control group [21,26]. Among studies with different comparators, one study showed non-significantly shorter duration compared to the previous version of an SDCE and one study showed non-significantly shorter duration with direct compared to indirect admission to an SDCE [24,25].

**Table 1:** Summary of the studies. ACE unit: Acute Care for Elders unit.

First Author	Year of Publication	Country	Type of Publication	Type of Intervention	Number of Subjects	Intervention Group	Control Group	Main Conclusions	Risk of Bias
Bleasdale [19]	1996	UK	Non-randomized controlled trial	Psychogeriatric unit	171	Delirious patients hospitalized in a specialist psychogeriatric ward	Delirious patients hospitalized in surgical and medical wards	The use of multidisciplinary guidelines for the management of delirium in hospital seems to improve outcomes. The guidelines were better followed on a specialist ward, but further work is needed to develop them and test their effectiveness in other centers.	Critical risk of bias
Eeles [22]	2013	Australia	Before–after study	Geriatric ward with delirium room	412	Delirious patients with agitation and/or high risk of fall	Delirious patients hospitalized in internal medicine	A dedicated unit for delirium management achieved a reduction in mortality. No significant improvement in length of stay, discharge destination or risk of falls.	High risk of bias
Goldberg [27]	2013	UK	Randomized clinical trial	Psychogeriatric unit	600	“Confused” patients hospitalized in psychogeriatric unit	“Confused” patients hospitalized in general ward	Specialist care for people with delirium and dementia improved the experience of patients and satisfaction of carers, but there were no convincing benefits in health status or service use.	High risk of bias
Lu [25]	2009	Australia	Retrospective cohort study	Psychogeriatric unit	142	Delirious patients admitted directly to psychogeriatric unit	Delirious patients admitted indirectly to psychogeriatric unit	Patients with delirium admitted directly to a secured co-located geriatric and psychogeriatric unit showed better outcomes and shorter LOS than those transferred from other wards.	High risk of bias
Flaherty [18]	2010	USA	Retrospective cohort study	Geriatric ward with delirium room	69	Delirious patients exposed to DR	Non-delirious patients hospitalized in geriatric ward	An ACE unit with a DR may improve ADL function from admission to discharge in patients with delirium compared with those without delirium. LOS and mortality were similar among patients with delirium compared with those without delirium.	High risk of bias
Flaherty [17]	2003	USA	Retrospective observational study	Delirium room	148	Delirious patients exposed to DR	No control group	The development of a DR, using basic geriatric principles on an ACE unit and key nursing interventions, represents a potential advance in the care of older delirious patients.	High risk of bias
Chong [21]	2014	Singapore	Randomized clinical trial	Delirium room	273	Delirious patients exposed to DR	Delirious patients hospitalized in geriatric ward	Compared to control subjects, DR patients showed significantly better results in functional improvement. No difference was found in the other outcomes.	High risk of bias
Chong [21]	2014	Singapore	Before–after study	Delirium room	320	Delirious patients exposed to DR	Delirious patients hospitalized before DR implementation	Compared to pre-DR, delirious patients showed improvement in delirium duration, length of stay, physical and chemical restraint, complication rate, and functional improvement. No difference in mortality and discharge destination was found.	High risk of bias
Mudge [26]	2013	Australia	Non-randomized controlled trial	Geriatric ward with delirium room	136	Patients with delirium or at-risk of delirium exposed to DR	Patients with delirium or at-risk of delirium not exposed to intervention	The intervention significantly reduced persistent delirium, reflecting better recognition of delirium by the care team. The intervention showed encouraging trends in reduction in mortality and falls.	High risk of bias
Wong Tin Niam [24]	2009	Australia	Before–after study	Delirium unit	228	Patients with possible delirium and agitation exposed to 2nd version of delirium unit	Patients with possible delirium and agitation exposed to 1st version of delirium unit	DR has the potential to improve patient care and outcomes.	High risk of bias

**Table 2:** Characteristics of the SDCE across study.

First Author	Number of Beds	Care Staff Intervention	Room Implementations	Pharmacological Interventions	Non-Pharmacological Interventions
Wong Tin Niam [24]	10	Nurse-to-patient ratio of 1:3.5 Video monitoring to nursing-doctors station Patient supervision guard for aggressive patients or at high risk of fall	Large clock and board for reorientation Familiar items including photographs	Protocol for pharmacological treatment to avoid indiscriminate, high-dose, and inappropriate use	Reorientation, routine establishment, and sleep hygiene Access to an activity room with an occupational therapy aid and access to a fenced garden area
Mudge [26]	4	Regular education sessions for all health team members Brochure provided for patients/carers	Removal of bedside cabinet Provision of chair and table for activities Clock and orientation board Appropriate lighting	Minimization of high-risk medications and judicious use of neuroleptic medication	Assistant in nursing trained in multisensory diversion and relaxation strategies Hospital volunteers for reorientation and cognitive activities Minimization of attachments
Chong [21]	5	Care delivery using staff trained in the person-centered care approach	An “elder-friendly environment”		Interventions addressing sensory impairments, dehydration, reality orientation, and early mobilization
					Management of sleep issues with light therapy
Flaherty [18]	4	One nurse station in the room with one nurse present 24h Slightly increased nurse:patient ratio (9.2 h/day vs. 8.2 h/day)			Minimization of noise sources and distractors Enhanced lighting Behavioral therapy and standard non-pharmacological approach No physical restraint
Flaherty [17]	4	Twenty-four hour nursing observation			Free of physical restraint Enhanced lighting Noise minimization Scrupulous application of geriatrics principles on delirium
Lu [25]	12	Close supervision with a staff desk with 270° glass paneling towards room and 24 h working camera Joint management by geriatricians and psychogeriatricians Nurse-to-bed ratio of 4.8h per patient day	Central activity/dining area		
Goldberg [27]	28	Additional staff specialized in mental health and a twice-weekly psychiatrist visit	Appropriate environment for people with cognitive impairment	Avoidance of psychotropic drugs when possible	Organized therapeutic and diversionary activities Proactive and inclusive approach to family carers Attention to nutrition, hydration, vision, hearing, and re-orientation Early mobilization and provision of activity. Avoidance of urinary catheters when possible
Eeles [22]	4	Increased nursing staff education on delirium Increased surveillance (hourly) of agitation and pain	Wall clock, orientation reminders, and patient biography Safer environment with low-profile beds, split rails and adjustable armchairs		Targeted nursing interventions (toileting, nutrition, diversion activity, mobility, reduced stimuli).
Bleasdale [19]	Not disclosed	Multidisciplinary team involvement Information and counseling given to carers	Creation of optimum environment (good lighting, not excessive sensory input) Orientation techniques (clock and calendar, familiar possessions)	Avoidance of major tranquilizers when possible	Avoidance of physical restraint when possible

#### 3.2.2. Length of Hospital Stay

Length of hospital stay was measured in nine studies [17,18,19,21,22,24,25,26,27] measuring the number of days spent in hospital. Five studies compared SDCEs with standard wards.

The results were inconsistent. Two studies reported a non-significantly longer length of stay, one reported a non-significantly shorter length of stay, one reported no difference, and one reported mixed findings, with a significantly shorter stay using a retrospective control group but a non-significantly longer stay using a prospective control group [19,21,22,26,27]. Among studies employing different comparators, one study showed significantly shorter length of stay compared with a previous version of SDCE, one reported a non-significantly shorter length of stay for direct versus indirect admission to SDCE, and one showed a non-significantly longer length of stay compared with non-delirious patients [18,24,25].

#### 3.2.3. Mortality

Mortality during hospital stay was measured in nine studies [17,18,19,21,22,24,25,26,27]. All studies assessed the proportion of patients who died during hospital stay. Five studies compared SDCEs with standard wards, four reported a non-significantly lower mortality rate in SDCEs, whereas one reported a non-significantly higher mortality rate [19,21,22,26,27].

Among the studies using different comparators, one study showed a non-significantly lower mortality rate compared with a previous version of SDCE, one reported a non-significantly lower mortality rate for direct versus indirect admission to SDCE, and one showed a non-significantly higher mortality rate compared with non-delirious patients [18,24,25].

#### 3.2.4. Fall Rate

The fall rate was assessed in six studies [21,22,24,25,26,27]. The fall rate was measured either as the proportion of patients who experienced a fall [21,22,24,26,27] or as the rate of falls per 1000 delirium-patient days [25]. Four studies compared SDCE with standard ward care: two reported a non-significantly lower fall rate, one showed a non-significantly higher fall rate, and one found no difference between groups [21,22,26,27].

Among the studies using different comparators, one study showed a non-significantly lower fall rate compared with the previous version of SDCE and one reported non-significantly lower fall rates for direct versus indirect admission to the SDCE [24,25].

#### 3.2.5. Physical Restraint Use

Physical restraint use was assessed in six studies [17,18,21,25,26,27]. Across all the studies, physical restraint use was measured as the proportion of patients who were physically restrained. Three studies compared SDCE with standard ward care: two studies reported no use of physical restraint in either group, whereas one reported significantly lower physical restraint use in the intervention group [21,26,27].

Among the studies using different comparators, one study showed a non-significantly lower rate of physical restraint use for direct versus indirect admission to the SDCE [25]. Two studies without a comparator group reported no use of physical restraints in the SDCE [17,18].

#### 3.2.6. Psychotropic Drug Use

Psychotropic drug use was assessed in six studies [17,19,21,25,26,27]. Psychotropic drug use was measured as the proportion of patients receiving chemical restraint in four studies [21,25,26,27] and as the proportion of patients who received a new chemical restraint during hospitalization in one study [19]. Four studies compared SDCE with standard ward care; two reported a non-significantly lower rate of psychotropic drug use, one reported a non-significantly higher rate of psychotropic drug use, and one showed mixed results, with a non-significantly higher rate when using a retrospective control group and a non-significantly lower rate when using a prospective control group [19,21,26,27].

Among the studies using different comparators, one study showed a non-significantly lower rate of psychotropic drug use for direct versus indirect admission to the SDCE [25].

#### 3.2.7. Functional Status

Functional status was assessed in four studies [17,18,21,26]. Functional status was measured as the proportion of patients experiencing functional decline in two studies, the proportion of patients demonstrating functional improvement and the mean improvement in Modified Barthel Index (MBI) score in one study, and the mean change in Activities of Daily Living (ADLs) in one study. Two studies compared SDCE versus standard ward care: one reported significantly greater functional improvement, whereas the other reported a non-significantly lower rate of functional decline in the SDCE group [21,26].

Among the studies using different comparators, one study showed non-significantly greater improvement in ADLs compared with non-delirious controls [18].

#### 3.2.8. Discharge Destination

Discharge destination was assessed in nine studies [17,18,19,21,22,24,25,26,27]. Discharge destination was measured as the proportion of patients discharged home in five studies [19,22,25,26,27] and as the proportion of patients discharged to the same level of care in two studies [21,24]. Five studies compared SDCEs with standard wards; all reported non-significant results favoring the intervention group [19,21,22,26,27].

Among the studies using different comparators, one reported no difference compared with a previous version of SDCE, one reported a non-significantly higher rate of discharge home for direct versus indirect admission to the SDCE, and one showed a non-significantly lower rate of home discharge compared with non-delirious patients [18,24,25].

#### 3.2.9. Professionals and Caregivers’ Satisfaction

Professionals and caregivers’ satisfaction was assessed in three studies [21,28,29]. One study explored family caregivers’ experience through interviews and highlighted the distressing experience associated with the onset of delirium in a close relative, characterized by feelings of sadness, shock, and concern about the future, alongside a substantial need for improved informational support. Care provided by professionals specialized in delirium offered some relief to caregivers [28]. A second study investigated healthcare professionals’ knowledge of delirium and found limited understanding of delirium prevention among hospital staff, as well as poor implementation of prevention guidelines. Despite this, staff expressed openness to further education and volunteer support in the care of patients with delirium [29]. The third study assessed family caregiver satisfaction with delirium care in the SDCE. In the intervention group, caregiver-reported satisfaction was high. Among interviewed caregivers, 86.6% considered the SDCE useful for recovery from delirium, and 83.5% considered the multicomponent intervention beneficial. Overall, 95.2% reported being satisfied or very satisfied with the care received, while 53.6% were satisfied with staff competence in managing their relative’s delirium [21].

No pooled analysis was conducted because each group (i.e., family and healthcare caregivers) was assessed in fewer than three studies.

### 3.3. Exploratory Pooled Analysis

Given the substantial clinical and methodological heterogeneity across studies, particularly with respect to intervention components and comparator groups, the pooled analyses should be interpreted as exploratory summaries of the direction and approximate magnitude of effects, rather than as definitive estimates of intervention efficacy. Overall, the direction of effect generally favored the intervention across outcomes. Compared with control groups, intervention groups showed higher odds of being discharged home or to the community, and lower odds of mortality and falls; however, these effects did not reach statistical significance. No clear difference was observed regarding the hospital length of stay. Functional improvement showed a small, non-significant effect favoring intervention, with substantial heterogeneity. The leave-one-out sensitivity analyses suggested that the direction of effect was generally stable for discharge destination, mortality, functional improvement, and falls. However, the precision and statistical significance of some estimates were influenced by individual studies. Hospital length of stay was less stable, as the exclusion of a study shifted the pooled estimate toward no effect and reduced heterogeneity. Overall, these findings warrant cautious interpretation, with greater emphasis on the consistency of effect direction than on definitive evidence of efficacy. Quantitative results are summarized in Figure 2 and Supplementary Table S2.

### 3.4. Sub-Group Analysis

No sub-analysis was carried out due to the lack of reporting on subgroups in the included studies.

### 3.5. Risk of Bias Assessment

All the studies were assessed to be at a high risk of bias according to Cochrane’s tools (Supplementary Figure S1).

## 4. Discussion

Despite the growing awareness of the high incidence and serious consequences of delirium in older hospitalized patients, there is still no consensus on the optimal standard of care. Current evidence supports multicomponent non-pharmacologic interventions as the first-line strategy for both prevention and management, yet considerable uncertainty remains regarding which specific types of intervention are most effective and how best to implement them in real-world settings. Within this context, dedicated specialized delirium care environments, such as delirium rooms, delirium units, and psychogeriatric and geriatric co-located units, have been proposed as an effective intervention to improve the implementation of multicomponent pharmacological and non-pharmacological interventions and therefore improve delirium care.

To our knowledge, this systematic review represents the first attempt to synthesize available evidence on these specialized care environments.

Regarding interventions’ characteristics, several studies proposed staff education about delirium’s definition, diagnosis, and management based on local or international guidelines [19,22,26,30]. A “senior-friendly” room design was proposed by most studies [19,21,22,24,25,26,27] even though specific descriptions were often lacking. Key characteristics included avoidance of architectural elements favoring falls, presence of adequate lighting, and presence of reorientation tools (clocks, calendars, personal belongings). Reduction in falling risk through higher surveillance [17,18,22,24,25], minimization of psychotropic drug use [19,24,26,27], reduction in physical barriers [21,22,26], and adequate lighting [19,26] were also proposed, as well as avoidance of physical restraint [17,18,19,21,26,27] and minimization of sensory overstimulation through appropriate environmental insulation and noise reduction [17,18,19,22].

In terms of additional environmental intervention, one study [21] evaluated the use of bright light therapy to improve sleep duration and quality, reporting promising results. This approach, together with other non-pharmacological interventions, such as music therapy [31], represents potential innovations in delirium care and warrants further investigation in the future.

The lack of precise, objective descriptions and the quantification of interventions suggests substantial heterogeneity in the interventions themselves, which may partly explain the high level of heterogeneity observed in study outcomes.

Regarding clinical outcomes, the substantial heterogeneity across the included studies precluded a formal meta-analysis aimed at estimating intervention efficacy. Instead, outcomes were described narratively, and an exploratory pooled quantitative analysis was conducted to summarize the direction and the approximate magnitude of effects. To reduce heterogeneity, this analysis was restricted to the subgroup of studies comparing SDCE with standard care in patients with delirium. Nevertheless, the resulting estimates should be interpreted with caution, as they do not allow causal inference regarding intervention effectiveness.

In addition, some pooled analyses relied on methodological assumptions, including the conversion of odds ratios to standardized effect sizes and the combination of multiple control groups. Although these approaches facilitated a broader quantitative synthesis, they may have introduced additional uncertainty and should be considered when interpreting the pooled results.

For delirium duration, the overall direction of findings was favorable. This is clinically plausible, as SDCEs are specifically designed to address several modifiable factors that may perpetuate delirium, as well as to improve recognition and monitoring of delirium.

Discharge destination was also largely positive, with over 75% of studies favoring intervention. This was particularly true for studies comparing SDCE with standard care, suggesting an intrinsic benefit of the SDCE, potentially through early mobilization, stronger rehabilitation, and inclusion of family caregivers in care.

Length of hospital stay was one of the most frequently assessed outcomes, yet results were mixed. Although shorter delirium duration could reasonably be expected to reduce hospitalization, length of stay is influenced by many factors beyond delirium itself, and overall longer hospital stays tend to take place when multidisciplinary care is applied, particularly in geriatric and psychogeriatric settings, compared to internal medicine and surgical wards. In addition, SDCEs may have preferentially received more behaviorally complex or clinically unstable patients in studies with no randomization, which could attenuate any beneficial effect on length of stay or even lead to apparently longer stays despite improved care.

Mortality showed a recurrent numerical trend in favor of SDCEs, but neither the individual studies nor the pooled analysis provided firm evidence of a statistically reliable effect. This pattern may reflect limited statistical power, since in-hospital mortality is a relatively infrequent event in several cohorts and the included studies were generally small or methodologically limited.

Falls were also not significantly reduced in any of the included studies, although several studies showed numerically lower fall rates in intervention groups. This outcome is particularly relevant because many SDCEs are explicitly designed to provide closer supervision, safer room layout, reduced use of restraints, and better behavioral management. However, falls are often under-reported in standard care, which might bias comparison, especially with retrospective control groups. Furthermore, in non-randomized studies, the preferential allocation of more agitated patients to SDCEs may have reduced the observed magnitude of intervention effects.

The findings on physical restraint use are particularly interesting from a care-quality perspective. Across all studies, physical restraint use was either absent or lower in SDCE than in comparator settings. This observation aligns with the core principle of specialized delirium care environments, which prioritize non-pharmacological de-escalation, close monitoring, environmental modifications, and structured therapeutic activity over restrictive or coercive measures.

Psychotropic drug use was heterogeneous; this finding is not surprising, as the outcome itself was inconsistently defined across studies, ranging from chemical restraint to the introduction of a new psychotropic drug after admission. Moreover, psychotropic drug prescribing for delirium is often reactive and strongly influenced by factors such as symptom severity, particularly hyperactive delirium, staff experience, local prescribing practice, perceived safety concerns, and availability of non-pharmacological alternatives. Some SDCEs may reduce psychotropic drug use through increased staff presence, enhanced environmental support, and structured non-pharmacological intervention. Conversely, others may increase the appropriate and targeted use of antipsychotic medications through improved recognition and management of severe behavioral symptoms. Therefore, the absence of a pooled effect does not necessarily imply that SDCEs have no influence on prescribing practice. Rather, it may reflect the fact that psychotropic drug use is a broad and highly context-dependent outcome that requires more precise operationalization to allow meaningful comparisons across studies.

Functional status outcomes were generally favorable, particularly in studies showing greater functional improvement or less decline in intervention groups, which is clinically plausible given the emphasis of SDCEs on early mobilization, stimulation, rehabilitation input, and avoidance of immobilizing practices.

The satisfaction findings add an important complementary perspective. Delirium is a highly distressing experience not only for patients but also for families and healthcare professionals, yet these aspects are often underrepresented in studies evaluating clinical effectiveness. The available qualitative and survey data suggest that caregivers value the specialized environment and perceive multicomponent interventions as beneficial to patients’ recovery. At the same time, these studies highlight substantial emotional distress and unmet informational needs among family members.

These findings support the notion that SDCEs may offer benefits extending beyond traditional biomedical outcomes, particularly through enhanced communication, emotional reassurance, and greater confidence in the quality and competence of care. Furthermore, they underscore the importance of incorporating patient-, family-, and staff-centered outcomes into future evaluations of delirium care models.

Taken together, these findings suggest that SDCEs are most likely to influence outcomes closely related to day-to-day delirium management, such as delirium duration, behavioral symptom management without the use of physical restraints, functional recovery, and discharge destination. In contrast, outcomes such as length of hospital stay and fall rate may be less responsive to these interventions or may be more strongly influenced by underlying patient characteristics, comorbidities, and organizational factors.

However, these findings should be interpreted with caution due to substantial heterogeneity across studies in intervention design, patient populations, and comparator groups, which limits causal inference. The review also has several limitations: only two randomized controlled trials and a small number of studies were included; all studies were rated as high risk of bias; and intervention models varied considerably, encompassing delirium rooms, delirium units, and psychogeriatric units with differing structures and support levels. Comparator groups were inconsistently defined and not always comparable to standard ward care. These factors reduce confidence in both the narrative and pooled findings. Future research should prioritize better-designed prospective comparative studies with clearer intervention definitions, more comparable control groups, and more standardized outcome measures. It would also be valuable to identify which specific components of specialized delirium care environments contribute most to any observed benefit. Future studies could explore whether more individualized non-pharmacological approaches, tailored to delirium subtype or patient profile, improve outcomes. Promising areas for further study may include interventions targeting circadian rhythm regulation, sensory stimulation, mobility, and other structured non-pharmacological strategies.

## 5. Conclusions

This review describes the available evidence on specialized delirium care environments for hospitalized older adults. Interventions were heterogeneous among studies, but they commonly included delirium-oriented staff training, enhanced observation, environmental adaptation, multidisciplinary care, and multicomponent non-pharmacological strategies. Reported outcomes were heterogeneous, with most outcomes showing a direction of effects favoring SDCEs, particularly for discharge destination, delirium duration, and mortality. Because the evidence base remains small, heterogeneous, and at a high risk of bias, these findings should be interpreted as a descriptive synthesis of existing care models and reported outcomes, rather than as evidence of efficacy. Further well-designed studies with clearer intervention descriptions, comparable control groups, and standardized outcome measures are needed to better understand how SDCEs can be implemented and evaluated in clinical practice.

## Data Availability

The original contributions presented in this study are included in the article/Supplementary Material. Further inquiries can be directed to the corresponding author.

## Supplementary Materials

The following supporting information can be downloaded at https://www.mdpi.com/article/10.3390/geriatrics11050122/s1. Figure S1: Cochrane risk of bias assessment; Table S1: Search strategies; Table S2: Pooled analysis quantitative outcomes; Table S3: Demographic data of the intervention group; Table S4: Demographic data of the control group.
