# Supplementary material for: Specialized Delirium Care Environments in Hospitalized Older Adults: A Systematic Review
Source: Geriatrics (Basel). 2026 Sep 4;11(5):122. doi: 10.3390/geriatrics11050122 (PMC13600139; doi:10.3390/geriatrics11050122)

Table S1. search strategies.

Database	Search string	Number of results
**Pubmed**	(delirium[Title/Abstract]) AND ("delirium room"[Title/Abstract] OR "delirium unit"[Title/Abstract])	16
**EMBASE**	delirium AND ("delirium room" OR "delirium unit" OR "geriatric monitoring unit")	32
**Cochrane Library**	delirium AND ("delirium room" OR "delirium unit" OR "geriatric monitoring unit")	2

Table S2.: Pooled analysis quantitative outcomes.

Outcome	k	Effect	95% CI	p value	Q	Heterogeneity p	I^2	Direction
Discharge home/community	5	OR 1.28	0.99 to 1.65	0.059	2.77	0.597	0.0%	Favours intervention
Hospital duration	4	SMD -0.06	-0.30 to 0.17	0.591	8.78	0.032	65.8%	Favours intervention
Functional improvement	3	SMD 0.22	-0.19 to 0.64	0.291	8.22	0.016	75.7%	Favours intervention
Falls	4	OR 0.76	0.26 to 2.16	0.600	5.32	0.150	43.6%	Favours intervention
Mortality	5	OR 0.55	0.30 to 1.03	0.061	7.05	0.133	43.3%	Favours intervention

Table S3. Demographic data of the intervention group. *Charlson score, mean ± SD.

INTERVENTION GROUP	Country	N of subjects	Age (mean + SD)	Sex (% female)	pre-exisiting dementia (% subjects)	living at home at admission (%)	% delirious patients (CAM+)	Functional status at admission	Co-morbidity status at admission	Diagnosis of delirium method	Type of delirium (% hyperactive)
**Bleasdale 1996 [19]**	UK	53									
**Eeles 2013 [22]**	Australia	237	80 + 10.4	51%						CAM	
**Goldberg 2013 [27]**	UK	310	85 + 80-88 (median + IQR)	55%	67%	72%	53%	9 + 5-13 (Barthel index, median + IQR)	4 + 3-5 (N of comorbities, median + IQR)	DRS	
**Lu 2009 [25]**	Australia	83	80.8 + 6.8	67.50%	55.40%	65.10%	100%		1.8 + 1.3	ICD-10	76%
**Flaherty 2003 [17]**	USA	69	82.6 + 6.3	65.50%	47.80%	71%	100%	5.6 + 3.6 (indipendent	2.1 + 1.2	ICD-9	

								scale 0-10, mean + SD)			
**Flaherty 2010 [18]**	USA	44	85.3 + 5.7	68%		34%	100%	4.1 + 4.6 (ADLs at admission, mean + SD)	2.7 + 2.2	modified CAM	
**Chong 2014 RCT [21]**	Singapore	234	84.1 + 7.4	56.40%	73.10%			28.8 + 24.1 (MBI, mean + SD)	2.2 + 1.6	CAM	51.70%
**Chong 2014 pre-DR [21]**	Singapore	234	84.1 + 7.4	56.40%	73.10%			28.8 + 24.1 (MBI, mean + SD)	2.2 + 1.6	CAM	51.70%
**Mudge 2013 [26]**	Australia	62	79.6 + 8.2	51.60%	51.60%	90.20%	30.60%	51.6% (indipendent in ADL pre-admission)		CAM	
**Wong Tin Niam 2009 [24]**	Australia	180	88 + 7	66%	58%	54%	70%				

Table S4. Demographic data of the control group. *Charlson score, mean ± SD.

**<u>CONTROL GROUP</u>**	**Country**	**N of subjects**	**Age (mean + SD)**	**Sex (% female)**	**pre-exisiting dementia (% subjects)**	**living at home at admission (%)**	**% delirious patients (CAM+)**	**Functional status at admission**	**Co-morbidity status at admission (Charlson score, mean + SD)**	**Diagnosis of delirium method**	**Type of delirium (% hyperactive)**
**Bleasdale 1996 [19]**	UK	118									
**Eeles 2013 [22]**	Australia	175	79.6 + 11.1	49%						CAM	
**Goldberg 2013 [27]**	UK	290	85 + 80-89 (median + IQR)	49%	63%	79%	62%	8 4-13 (Barthel index, median + IQR)	4 3-6 (N of comorbities, median + IQR)	DRS	
**Lu 2009 [25]**	Australia	59	79.3 + 7.3	54.30%	61%	71.20%	100%		2.1 (1.8)	ICD-10	66%

**Flaherty 2003 [17]**											
**Flaherty 2010 [18]**	USA	104	83.2 + 7.1	73%		46%	0%	7.4 + 4.7 (ADLs at admission, mean + SD)	2.8 (2.0)	modified CAM	
**Chong 2014 RCT [21]**	Singapore	39	84.5 + 8.2	64.10%	53.80%			31.6 + 28.8 (MBI, mean + SD)	2.3 + 1.3	CAM	51.30%
**Chong 2014 pre-DR [21]**	Singapore	47	84.3 + 9.2	80.90%				39.5 +33.6 (MBI, mean + SD)	3.6 + 2.2	CAM	
**Mudge 2013 [26]**	Australia	74	82.3 + 7.7	49%	56.80%	79.70%	36.40%	45.9% (independent in ADL pre-admission)		CAM	
**Wong Tin Niam 2009 [24]**	**Australia**	**48**	**85 + 8**	**65%**	**60%**	**83%**	**35%**				

Figure S1. Cochrane Risk of Bias assessment.

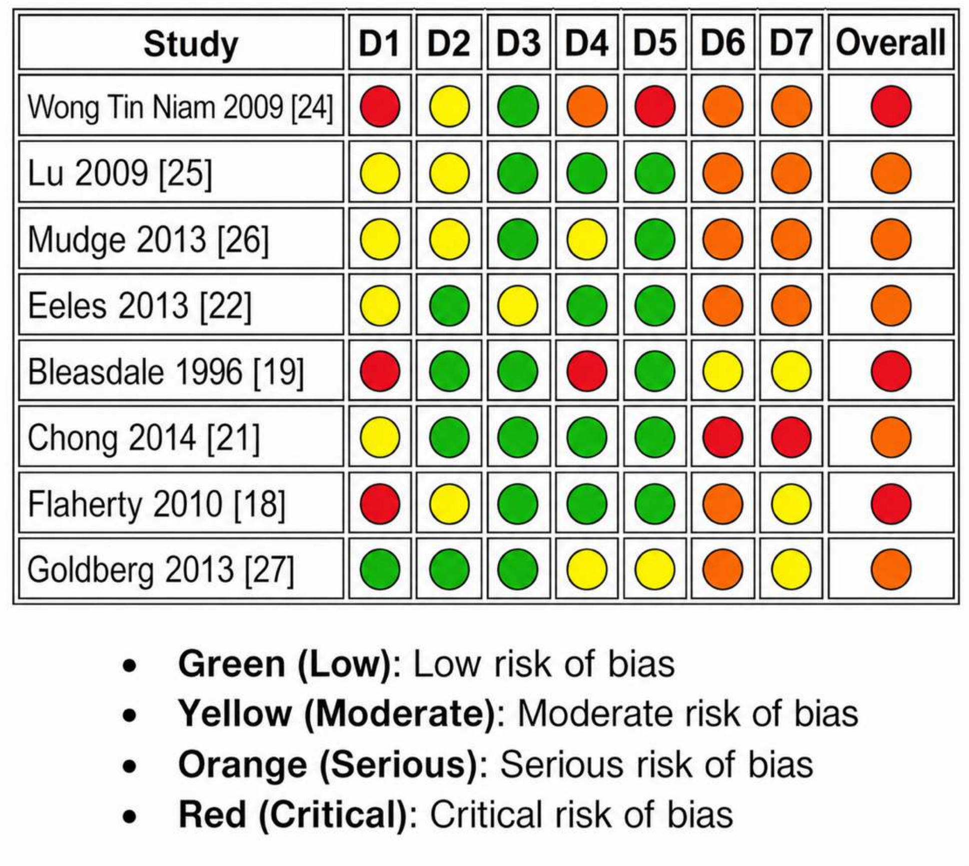

Supplement: Supplementary file 1 [file geriatrics-11-00122-s001.zip › geriatrics-4307522-supplementary.pdf]
